# Sexuality following trauma injury: A literature review

**DOI:** 10.4103/2321-3868.130189

**Published:** 2014-04-06

**Authors:** Kylie Marie Connell, Rosemary Coates, Fiona Melanie Wood

**Affiliations:** 1 Department of Sexology, School of Public Health, Faculty of Health Sciences, Curtin University, GPO Box U1987, Perth, Western Australia Australia; 2 Fiona Wood Foundation, Perth, Western Australia Australia; 3 Royal Perth Hospital, Burns Service of Western Australia, Western Australia Australia; 4 Burn Injury Research Unit, University of Western Australia, Perth, Western Australia Australia

**Keywords:** Burns, rehabilitation, spinal cord injury, sexuality, traumatic brain injury

## Abstract

Restoration of the quality of life (QoL) of trauma injury survivors is the aim of trauma rehabilitation. It is generally acknowledged that sexuality is an important component of QoL; however, rehabilitation services frequently fall short of including sexuality as a matter of routine. The literature was reviewed to examine the experiences of trauma survivors from three groups: spinal cord injury (SCI), traumatic brain injury (TBI) and burns. The focus was on the impact of trauma on the QoL to identify future research directions and to advocate for the inclusion of sexuality as an integral part of rehabilitation. Databases searched were Proquest, Ovid, Cinahl, Medline, PsycInfo and Cochrane Central Register of controlled trials. A total of 36 eligible studies were included: SCI (*n* = 25), TBI (*n* = 6), burns (*n* = 5). Four themes were identified across the three trauma groups that were labeled as physiological impact of trauma on sexuality, cognitive-genital dissociation (CGD), sexual disenfranchisement (SD) and sexual rediscovery (SR). Trauma injury has a significant impact on sexuality, which is not routinely addressed within rehabilitation services. Further sexuality research is required among all trauma groups to improve rehabilitation services and in turn QoL outcomes for all trauma survivors.

## Introduction

Improved rate of survival post-trauma has precipitated a shift from outcomes measured in terms of mortality to outcomes being measured in terms of activities of daily living (ADLs) and quality of life (QoL).[1,2]

Sexuality is a component of many of the QoL domains; however, current QoL measures do not adequately incorporate sexuality within their assessment components, thus perpetuate the exclusion of sexuality as a matter of discourse within trauma rehabilitation.[3]

**Table:** 

Access this article online	
**Quick Response Code**:	**Website**: www.burnstrauma.com
	**DOI**: 10.4103/2321-3868.130189

The incidence and prevalence of sexuality issues for trauma survivors is impossible to estimate due to a lack of consistency within sexuality research with regard to sexuality domain definitions, data collection methods, and measurement tools. Phelps *et al.*,[4] study of married or partnered men with a spinal cord injury (SCI) suggested that while 96% of participants were sexually active, 20% reported little to no sexual desire and 44% reported dissatisfaction or indifference with their sexual relationship. Mendes *et al.*,[5] in their study of sexual satisfaction in men with SCI found that 60% of their participants were satisfied with their sexual lives. However, their findings did indicate that there was a significant fall in sexual satisfaction following SCI with 80% (32 males) in the prelesion period of the study reporting to be very satisfied with this dropping to 47.5% (19 males) in the postlesion study period. There has been little empirical research conducted regarding female sexuality following SCI. Kettl *et al.*,[6] study of female sexuality following SCI found that 26% of participants rated sex as less important following injury and 23% were less satisfied with their sexual lives. Most significantly, this study found that the majority of women experienced body image changes with 52% reporting that their bodies were less attractive than they were before their SCI. The authors suggest that these body image changes may play a role in both the reduction of sexual activity and enjoyment found in the study.

Changes to sexual functioning have also been documented following brain injury. In Kreuter *et al.*,[7] study of males and females post-traumatic brain injury (TBI), 56% of the participants (*n* = 92) reported dissatisfaction with the frequency of sexual activity with 31% identifying a lack of intimate partner being the main contributing factor. In contrast, 86% of the respondents who had an intimate partner were satisfied with their sexual lives and 75% were satisfied with the frequency of intercourse. Ponsford[8] study also supported these findings with 54% of participants (*n* = 280) reporting a decrease in sexual frequency. In addition, 41% expressed a decrease in sexual drive, 39% felt the ability to satisfy their partner had decreased, 38% had a decreased ability to engage in sexual intercourse, and 38% reported less enjoyment in sexual activity. The author concluded that more than 50% of people following a TBI experience significant sexual changes up to 5 years post-injury.

There has been very little research regarding sexuality following burn injuries, thus research involving adequate methods and sample sizes are scarce. Evidence from a western Australian burn unit sample suggests that burn injuries have a significant impact on sexuality and body image satisfaction and that these changes are potentially long standing. [9,10] Our preliminary data analysis of responses from the Burns Specific Health Scale-Brief suggests that over 20% of burn survivors (*n* = 362) report a loss of sexual interest up to 12 months post-injury.[9] Body image dissatisfaction is high among burn survivors with over 40% indicating the perception that they are unattractive to others and 40% indicating that their scars bother them.[9] A significant finding was that the severity of the responses, as scored via a Likert scale, was much higher in females especially for the body image domains.[9] We concluded that sexuality and body image may have a role in post-burn psychosocial adjustment and QoL that requires further research.[9]

Sexuality is a dynamic entity in which the interplay of biological, psychological, sociological, spiritual and cultural factors influence personality development sense of self-esteem and ability to form interpersonal relationships.[11,12] In order to conceptualize the dynamics involved, the World Health Organization’s (WHO) (2002) working definition of sexuality[13] will be used as a frame of reference when using the term sexuality throughout this review.

The research question guiding this literature review was as follows: What are the experiences of adult trauma survivors regarding how changes in their sexuality impact their QoL? SCI, TBI and burn trauma groups were chosen for this review on the basis that they represented common trauma diagnoses and provided a wide range of biopsychosocial rehabilitation issues in which to compare.

## Methods

### Search strategy

A search was conducted up to March 2013 using ProQuest, Ovid, CINAHL, PubMed, and Medline databases. SCIs, TBIs, acquired brain injury, burns and disfiguring scarring were used as the free text word searched with sex^1^, sexual^2^, sexual adjustment, sexual satisfaction and sexual rehabilitation as key words.

### Eligibility criteria

Research articles published between January 1990 and January 2013 pertaining to sexuality following SCI, TBI and burns were included within this review to look at current issues in sexual rehabilitation.

Both quantitative (QUAN) studies that evaluated sexual changes through outcome measures and qualitative (QUAL) studies that investigated the ‘lived experience’ of sexuality changes in adult trauma survivors were included in this review.

Research articles were included if they described research in which sexuality of the individual trauma groups was investigated. Case studies were initially excluded from the eligibility criteria, as they provided anecdotal information only. However, due to low numbers of research articles regarding sexuality following burns and TBIs compared with SCI literature, they were subsequently included for these 2 trauma groups to provide potential insight regarding sexuality issues to compare with the SCI literature.

### Exclusions

Reviews, opinion pieces, expert opinions and papers that did not deal specifically with the 3 trauma groups were excluded. Research articles focusing solely on the physiological aspects of sexual function were eliminated; however, research regarding sexual functioning that encompassed the broader aspects of sexuality as outlined by the WHO’s (2002) working definition of sexuality[13] were included.

### Data abstraction

In order to analyze qualitative data within the literature, themes were identified and labeled to attribute meaning to phenomena.[14] Themes were further refined according to the importance of the theme based upon the frequency that the phenomena appeared throughout the literature, the significance of the phenomena as identified by the research articles, and the theme’s pervasiveness across the trauma groups selected for review and their rehabilitation services.[15]

A phenomenological study conducted by Tepper *et al.*,[16] of sexual experiences of women following SCI identified common post-injury psychosocial phenomena, which were grouped and labeled as ‘cognitive-genital dissociation’, ‘sexual disenfranchisement’, and ‘sexual rediscovery’. These labels were used as they were relevant to understanding the psychosocial components of the body of literature reviewed in relation to sexuality post-burn, TBI and SCI trauma groups, as opposed to SCI alone. In addition to these 3 themes, a fourth theme emerged regarding the physiological impact of trauma on sexuality, and thus is also included in this review.

## Results

Publication titles and abstracts were reviewed online. A total of 36 eligible studies were identified for review: SCI (*n* = 25), TBI (*n* = 6), and burns (*n* = 5) [Table 1].

**Table 1:** **Articles included in review**

Author(s)	Title	Participants (*n*)	Methods	Sexuality issues addressed
SCI Articles				
Anderson *et al.*, (2007)	Spinal cord injury influences psychogenic as well as physical components of female sexuality	87 (f)	General questionnaire*	Sexual response
				Sexual satisfaction
Anderson *et al.*, (2007)	Long-term effects of spinal cord injury on sexual function in men: Implications for neuroplasticity	199 (m)	General questionnaire*	Sexual response
				Sexual satisfaction
Cardoso *et al.*, (2008)	Self-awareness of the male sexual response after spinal cord injury	90 (m)	General questionnaire*	Sexual response
Ferreiro-Velasco *et al.*, (2005)	Sexual issues in a sample of women with spinal cord injury	37 (f)	Semi structured interview	Sexual response
				Sexual satisfaction
				Sexuality and rehabilitation
Fisher *et al.*, (2002)	Sexual health after spinal cord injury: A longitudinal study	32 (m) 8 (f)	The Sexual Health Needs Survey*	Sexual activity
				Sexual adjustment
				Sexual satisfaction
				Sexuality and rehabilitation
Forsythe and Horsewell (2006)	Sexual rehabilitation of women with a spinal cord injury	70 (f)	Discussions from workshops	Sexuality and rehabilitation
Hess *et al.*, (2007)	The experience of four individuals with paraplegia enrolled in an outpatient interdisciplinary sexuality program	4 (m)	Sexual History Questionnaire*	Sexuality and rehabilitation
Kettl *et al.*, (1991)	Female sexuality after spinal cord injury	27 (f)	Descriptive questionnaire*	Sexual satisfaction
				Sexual response
				Sexuality and rehabilitation
Kreuter *et al.*, (2008)	Sexuality and sexual life in women with spinal cord injury: A controlled study	532 (f)	Spinal Cord Injury Women Questionnaire*	Sexual activity
				Sexual response
				Sexuality and rehabilitation
Kreuter *et al.*, (1996)	Sexual adjustment and quality of relationships in spinal paraplegia: A controlled study	64 (m) 11 (f)	Relationship questionnaire*	Sexual activity
			Quality of Life Visual analogue	Sexual response
			Hospital Anxiety and Depression Scale (HAD)	Sexual satisfaction
Leibowitz (2005)	Sexual rehabilitation services after spinal cord injury: What do women want?	24 (f)	Semi structured interviews	Sexual activity
				Sexuality and rehabilitation
Leibowitz and Stanton (2007)	Sexuality after spinal cord injury: A conceptual model	24 (f)	Semi structured interviews	Sexual activity
				Sexual response
				Sexuality and rehabilitation
Lysberg and Severinsson (2003)	Spinal cord injured women’s views of sexuality: A Norwegian survey	48 (f)	Descriptive questionnaire*	Sexual satisfaction
Mendes *et al.*, (2008)	Sexual satisfaction in people with spinal cord injury	90 (m) (40 with SCI, 50 without SCI)	Descriptive questionnaire*	Sexual response
				Sexuality and rehabilitation
Li & Yau (2006)	Sexual issues and concerns: Tales of Chinese women with spinal cord impairments	10 (f)	In-depth interviews	Sexual activity
				Sexuality and rehabilitation
Parker and Yau (2012)	Sexuality and women with spinal cord injury	4 (f)	Semi-structured interviews	Sexual adjustment
				Sexuality and rehabilitation
Phelps *et al.*, (2001)	Spinal cord injury and sexuality in married or partnered men: Activities, function, needs, and predictors of sexual adjustment	50 (m)	Multiple choice questionnaire*	Sexual response
				Sexual satisfaction
				Sexual adjustment
				Sexuality and rehabilitation
Potgieter and Khan (2005)	Sexual self-esteem and body image of South African spinal cord injured adolescents	4 (m) 3 (f)	Descriptive questionnaires*	Sexual adjustment
			In-depth interviews	
Reitz *et al.*, (2004)	Impact of spinal cord injury on sexual health and quality of life	47 (m) 16 (f)	Descriptive questionnaire*	Sexual activity
				Sexual satisfaction
				Sexual adjustment
Richards *et al.*, (1997)	Women with complete spinal cord injury: A phenomenological study of sexuality and relationship experiences	15 (f)	Semi-structured interviews	Sexual response
				Sexual activity
				Sexual adjustment
				Sexuality and rehabilitation
Singh and Sharma (2005)	Sexuality and women with spinal cord injury	40 (f)	Interview questionnaire*	Sexual response
				Sexuality and rehabilitation
Tepper *et al.*, (2001)	Women with complete spinal cord injury: A phenomenological study of sexual experiences	15 (f)	Semi-structured interviews	Sexual response
				Sexual activity
				Sexual adjustment
				Sexuality and rehabilitation
Westgren and Levi (1999)	Sexuality after injury: Interviews with women after traumatic spinal cord injury	8 (f)	Structured interviews	Sexual activity
				Sexual concerns
				Sexuality and rehabilitation
Westgren *et al.*, (1997)	Sexuality in women with traumatic spinal cord injury	62(f)	Structured interviews	Sexual activity
			Descriptive questionnaire*	Sexual satisfaction
			Descriptive questionnaire*	
White *et al.*, (1993)	Sexual activities, concerns and interests of women with spinal cord injury living in the community	40 (f)	Functional Independence Measure (FIM)	Sexual activity
				Sexuality and rehabilitation
TBI Articles				
Dombrowski *et al.*, (2000)	Rehabilitation treatment of sexuality issues due to acquired brain injury	2 (m) 1 (f)	Case studies	Sexual activity
				Sexual behaviour
				Sexuality and rehabilitation
Gaudet *et al.*, (2001)	Self-reported consequences of traumatic brain injury: A study of contrasting TBI and Non-TBI participants	26 (m TBI) 24 (f TBI), 22 (m non-TBI) 33 (f non-TBI)	Descriptive questionnaires*	Sexual satisfaction
				Sexual adjustment
Kreuter *et al.*, (1998)	Sexual adjustment and its predictors after traumatic brain injury	65 (m) 27 (f)	Descriptive questionnaire*	Sexual response
				Sexual activity
				Sexual satisfaction
O’Carroll *et al.*, (1991)	Psychosexual and psychosocial sequelae of closed head injury	30 (m) 6 (f)	Golombok Rust Inventory of Sexual Satisfaction (GRISS)	Sexual response
			General Health Questionnaire	Sexual activity
			Hospital Anxiety and Depression Scale (HAD)	Sexual satisfaction
Ponsford (2003)	Sexual changes associated with traumatic brain injury	143 (m) 65 (f)	Descriptive questionnaire*	Sexual response
				Sexual activity
				Sexual adjustment
				Sexuality and rehabilitation
Sandel *et al.*, (1996)	Sexual functioning following traumatic brain injury	39 (m) 13 (f)	Derogatis Interview for Sexual Function (DISF) Neuropsychological measures	Sexual response
				Sexual activity
			Neuropsychological measures	Sexual adjustment
Burn Articles				
Dobkin de Rios *et al.*, (1997)	Sexual dysfunction and the patient with burns	4 (m) 1 (f)	Case Reports	Sexual response
Bianchi (1997)	Aspects of sexuality after bur injury: Outcomes in men	40 (m)	The Sexuality Scale	Sexual adjustment
Connell *et al.*, (2012)	Sexuality following burn injuries: A preliminary study	268 (m) 94 (f)	Burns Specific Health Scale-Brief Version	Sexual adjustment
				Sexuality and rehabilitation
Meyer *et al.*, (2011)	Sexual attitudes and behaviour of young adults who were burned as children	50 (m) 42(f)	What Young People Believe and Do Questionnaire	Sexual activity
				Sexual adjustment
Parrott and Esmail (2010)	Burn survivors’ perceptions regarding relevant sexual education strategies	Phase 1: 3 (m) 5 (f)	Semi-structures interviews	Sexual adjustment
		Phase 2: 3 (m) 1 (f)	Focus group	Sexuality and rehabilitation
Robert *et al.*, (1998)	Impact of disfiguring burn scars on adolescent sexual development	10 (m) 9 (f)	What Young People Believe and Do-Revised (WYPBD)	Sexual activity
				Sexual adjustment

*Denotes questionnaires developed by the author for the purposes of their study and thus not validated. (m) = males (f) = females. Gender labels male and female were used as this is how gender was represented within the literature. SCI = Spinal cord injury, TBI = Traumatic brain injury

### Physiological effects of trauma on sexuality post-trauma injury

Physiological issues affected sexuality across all 3 trauma groups with sexual function, pain, medication side effects, and decreased desire/libido being the most common factors. Gender comparisons have not been made due to the lack of gender specific sexuality research.

Decreases in sexual arousal and desire were common factors across most of the trauma groups reviewed.[4,5,7,8,17–24] While high rates of difficulties in sexual arousal correlated with decreases in sexual satisfaction, most studies did not include definitions of sexual arousal or sexual desire, and as such, it is difficult to ascertain which components of these processes have the greatest potential impact. Understanding concepts of sexual desire and the processes of sexual arousal in both males and females are important, not only to understand the physiological changes that may occur post-trauma injury but also to understand the impact of treatment and rehabilitation methods and identify future research directions.

Psychotropic medications commonly used in the treatment of post-traumatic stress disorder (PTSD) and depression following trauma injuries have been noted to have secondary sexual dysfunction side effects.[25] Antidepressant medications, particularly selective serotonin reuptake inhibitors (SSRIs), have clinical implications that include pharmacological sexual dysfunction.[26] There were no conclusive studies relating to PTSD and sexual function in the 3 trauma groups selected, which included pharmacological prescription. Improvements in PTSD symptomology (e.g. depression) following sexual therapy have been noted in burn survivor rehabilitation, with these improvements occurring regardless of the implementation of psychopharmalogical intervention, suggesting that pharmalogical interventions played only a small part in the triggering of sexual dysfunction in this population.[25]

Difficulties relating to sexual function including erectile or ejaculatory function, lubrication,[4,5,17,24,27,28] and orgasm difficulties[5,6,21–24,28] were widespread within the trauma literature, particularly for SCIs. SCI literature identified that chronic pain and spasticity have been linked to increases in depression symptomology, decreased sexual arousal, and impaired erectile ability resulting in pain being associated with a negative impact on sexual function.[24] Pain was noted as a common reason for why SCI survivors avoided engaging in sexual activity.[23,27]

Bladder and bowel incontinence during sexual activity was among the most significant areas of concern for people with SCI and the associated embarrassment of this issue lead to sexual and relationship avoidance in this trauma group.[12,17,22,27,29–31] While there are many strategies for dealing with bladder and bowel management for sexual activity to reduce embarrassment for individuals, research suggests that addressing issues was significantly influenced by personal comfort levels of individual health professionals.[3] While medical and allied health staff may be comfortable dealing with genitalia in relation to personal care issues such as continence management, comfort levels decrease when the personal care issue is based upon sexual pleasure, leaving sexuality largely ignored.[3,16,31,32]

### Cognitive-genital dissociation (CGD)

The process of “shutting down” or “shutting out” of sexuality based on the internalized perception that following trauma injury individuals are no long capable of giving or receiving sexual pleasure[16,33] was intrinsically linked within the literature with the exclusion of accurate, informative, and timely sexuality-related information and interventions within the rehabilitation setting,[7,12,16,33,34] from sexuality-specific trained staff.[35] Internalizing inaccurate self-beliefs that a mutually satisfying and pleasurable sexual relationship post-injury is not possible lead to greater rates of depressive symptomology and decreases in overall QOL within the trauma literature.[4,21,27,36]

Potgieter and Khan[37] described a dissociative process where individuals view their altered physical body as a dual entity following SCI. This process is characterized by individuals experiencing positive feelings toward their individual body parts while suppressing or dissociating their feelings toward their altered or impaired body. The result of this process is the perception of the altered body as an ‘enemy’. Decreases in sexual esteem and satisfaction were found to have strong correlations with greater rates of depression, anxiety and PTSD symptomology among the trauma groups reviewed.[6,16,33,34,37] The exclusion of accurate sexuality information and resources from rehabilitation services contributed to sexual difficulties, which had a direct impact on poor sexual esteem, sexual satisfaction and body image.[6,16,31,33,38,39] Individuals internalizing that they are no longer desirable or capable as a sexual partner was a significant factor in high rates of sexual avoidance behavior amongst most trauma group survivors reviewed.

The importance of including sexuality within rehabilitation programs was reiterated throughout the literature for all trauma groups, as well as, an overall dissatisfaction with how sexuality is currently addressed. In general, research indicated that trauma survivors receive little or no advice from health professionals regarding potential direct and indirect changes to their sexuality as a result of their injury.[8,16,27,33] This results in little or no avenues for trauma survivors to address sexuality issues, such as normalizing insecurities and alleviating fears regarding changes to one’s sexuality.

There was anecdotal evidence that standard sex therapy techniques, such as sensate focus, can facilitate significant improvements in QoL and intimate partner relationships in burn survivors.[25] Four potential factors have been defined that may contribute to sexual dysfunction following a burn injury: psychopathology factors (the presence of adjustment problems including PTSD and depression symptomologies), psychodynamic factors (changes in body image and self-esteem affecting arousal), medication side effects (sexual side effects of antidepressants on sexual function and desire), and surgical and pain factors (the functional effects of contractures and tissue damage as well as the effects of chronic pain on arousal).[25] Unfortunately, little or no empirical evidence to recommend the styles of therapy, timing of intervention, who is best to provide this intervention, and how it should be initiated with patients has been conducted extensively within any of the trauma group literature reviewed. However, there is acknowledgement within some literature for including health professionals specializing in sexuality in rehabilitation programs due to the complexities involved in sexual therapy and rehabilitation.[20,31]

### Sexual disenfranchisement (SD)

Decreased body image was a common phenomenon throughout the trauma research articles. Body image issues were generally seen as a complex issue, with a possible stronger impact on female QoL outcomes, due to the perceived greater importance of internalized stereotypes of concepts of beauty on female self-esteem.[7,12,18,27]

The disconnection and disappointment associated with SD may result in individuals avoiding engagement in sexual activity with their partners due to the internalized belief or experience that sex is less satisfying post-injury.[33] This may result in a loss of sexual self-esteem and confidence in the ability to experience sexual pleasure in an enjoyable way following trauma.[16] Exclusion of sexuality from rehabilitation services, lack of sexual counseling, asexual societal attitudes regarding people with disabilities, body image concerns, and negative feedback regarding sexuality from health professionals and partners may also contribute to the experience of SD.[16,33] In general, there were no correlations between the type of injury or injury severity with decreases in sexual satisfaction, sexual self-esteem, or frequency of sexual activity, suggesting that trauma injury can have a major impact on an individual’s sexuality, body image and interpersonal relationships, regardless of the nature of the trauma or length of time post-trauma.

The negative impact of societal stereotypes on sexual self-esteem was evident across the 3 trauma groups reviewed. Internalizing stereotypical scripts that people with disabilities are asexual, undesirable[16] and the perceived taboo of an ‘able’ bodied person having a sexual relationship with a ‘disabled’ person, can have a negative impact on body image, sexual arousal[17,35] and depression symptomology.[35]

Body image dysphoria (BID) studies in non-trauma populations emphasize a multidimensional concept of body image that includes perceptual (accuracy of appearance-related evaluations), subjective (levels of dissatisfaction, anxiety, or distress in relation to one’s appearance) and social-behavioral (discomfort and avoidance of activities or situations that may bring attention to an individual’s appearance issues) facets.[40] Connections between negative body image perception and psychosocial maladjustment were found within the trauma literature.[17,27,37,41,42] Scarring, disfigurement, deformity and loss of function, which are characteristics of many trauma injuries, were found to lead to perceptual, subjective and social body image changes, resulting in decreases in body image satisfaction and behavioral and social avoidance.[9,16,33] Evidence also suggests that this process is greater in females, who potentially place greater importance of normative social scripts of beauty based on media influences, on the subjective evaluations of oneself.[37]

Research regarding severity of trauma and functional limitations on an individual’s sexuality and relationship development are limited and contradictory. Several studies across the trauma groups indicate against popular thought that there is no significant correlation between injury severity as a predictor of psychosocial and sexual adjustment.[1,2,19,41,43–45] For example, injury severity in terms of level of injury and completeness of spinal lesion has not been found to correlate to decreases in sexual satisfaction,[5] sexual adjustment, and quality of relationships post-SCI,[41] thus decreases in these areas have been found regardless of injury severity. However, evidence within burn literature has found there is a correlation between impact of injury severity, as measured by the percentage of total body surface area (TBSA) burnt, and decreased sexuality and body image satisfaction have been found in both males and females.[9]

### Sexual rediscovery (SR)

SR has been characterized by an increase in sexual self-esteem,[16] where there is an improved confidence in the ability to communicate one’s own sexual needs with a partner.[33] Sexual experimentation was synonymous with renewed sexual interest and exploration. Willingness for both partners to explore a varied sexual repertoire and the corresponding perception that one’s partner enjoyed the sexual component of the relationship were found to be important correlates of positive sexual experiences and satisfaction in trauma survivors.[41] Exploring and developing a varied sexual repertoire as an integral component of SR and satisfaction was echoed throughout SCI and sexuality research.[12,17,21,27,32] Common examples found within the literature of variations to sexual activity were exploration and stimulation of erogenous zones throughout the body,[17,27,41] incorporating sexual aids/toys,[12,27,41] incorporating fantasy,[12,22,27] using the senses,[27] and incorporating erotic or pornographic media.[12] In addition, spending time with one’s partner participating in non-genitally focussed intimacy, e.g. holding hands and hugging and kissing were important factors in relationship and sexual satisfaction overall.[27,32,45]

## Discussion

Broadening the definitions of sexuality within the rehabilitation settings away from genitally focussed influences, to incorporate intimacy, pleasure and variability sexual expression, would assist sexual adjustment.[31,32,41]

Timing of addressing issues of a sexual nature is a crucial consideration within rehabilitation treatment planning.[27] Trauma survivors may initially experience CGD, whereby sexual exploration does not necessitate functional ADL tasks essential for discharge from hospital and reintegration to the home and workforce.[16] As such the initial phases of rehabilitation may be too early to comprehensively address issues of changes in sexuality, thus it is essential that sexuality services be available on an ongoing basis as part of outpatient services.[27,31] Post-discharge, individuals may develop a greater understanding of their physical and sexual changes once discharged, which can be addressed more realistically within outpatient sexual counseling and rehabilitation services,[31] provided the patient can still access these services overtime.

Sexual medicine and rehabilitation is concerned with sexuality changes, dysfunction, or problems in people who have a congenital or acquired physical impairment due to disease, injury, or secondary to medical interventions.[46] The article by Hatzichristou *et al.*,[47] outlining recommendations for the clinical evaluation of men and women with sexual dysfunctions identifies three basic principles for the management of sexual problems in the clinical setting. These principles are 1) the adoption of a patient-centered framework, with emphasis on cultural competence in medical practice (taking into account biopsychosocial factors in accordance with the WHO’s definitions of health)[13], 2) application of evidence-based medicine in diagnostic and treatment planning (assessment and treatment should be guided by best evidence-based research and practice), and 3) use of a unified management approach in evaluating and treating sexual problems in both men and women (this is described as incorporating the need for gender equality in sexual medicine, although the authors of this review suggest this should include further expansion of concepts of gender and diverse sexual orientations). While these guidelines have been described in terms of sexual medicine, it provides a potential framework for working with sexuality changes that can be adapted to the multidisciplinary healthcare setting.

Hatzichristou *et al.*,[47] classify sexual dysfunctions as 1) psychogenic (the absence of biological findings, 2) organic (biological findings, although not significant mental/cognitive or emotional/affect distress), and 3) mixed (biological findings with significant mental/cognitive or emotional/affect distress). The authors stress that organic and psychogenic factors are often both present, particularly for those with long-standing sexual function problems. An alternative classification of sexual dysfunction following trauma or chronic illness, i.e. potentially better suited for a multidisciplinary clinical context, is that described by Foley and Gimbel.[48] They describe 3 levels of sexual changes for people with multiple sclerosis—primary, secondary, and tertiary—although it is our belief that these levels of sexual changes could apply to all trauma and chronic illness groups. Primary sexual changes relate to nervous system damage that impairs physiological aspects of sexual response, e.g. decreases in sexual desire, painful or unpleasant genital sensations, and changes to frequency and/or intensity of orgasmic response. Secondary changes relate to other symptoms and/or physical limitations that indirectly impact sexual response, e.g. difficulties with bowel and bladder continence during sexual activity, fatigue and muscle weakness limiting body positioning. Lastly, tertiary sexuality changes result from psychosocial and cultural issues that can have a negative impact on sexual esteem, body image and sexual satisfaction, e.g. changes in roles, lifestyle, and participation in social, work, or leisure activities. Tertiary sexual changes may also be linked with greater rates of depression, decreased self-esteem and decreased body image for people with disabilities[16,49,50]

While any classification system of health-related issues should be utilized with caution and/or care, they do provide a potential framework in which the combination of biopsychosocial issues can be factored into screening within the clinical setting for assessment and treatment planning. Unfortunately, there were no individual or multicenter clinical trials of screening or assessment tools of sexuality changes for use by health professionals in any of the trauma group literature included in this review. This finding highlights a significant gap in evidence-based knowledge and practice for all trauma diagnostic groups that require further attention with empirical research.

Of the 36 eligible articles included in this review, only 3 included a validated sexuality-related outcome measure. The 3 measures that were included within the literature reviewed were all designed primarily for research use and are potentially not suited for the multidisciplinary clinical setting. In addition, there have also been some construct validity issues identified with ‘The Sexuality Scale’ used in one of the studies.[51]

There are scales available that could be considered for future research and clinical practice in the area of sexuality and trauma injury. The Female Sexual Function Index (FSFI) developed by Rosen *et al.*,[52] was designed as a brief, valid, and reliable self-reported measure for use as clinical trials assessment instrument. The authors state that the FSFI addresses the multidimensional nature of female sexual function based on 5 factors/domains: a) desire and subjective arousal, b) lubrication, c) orgasm, d) satisfaction and e) pain and/or discomfort.[52] The FSFI is a 19-item self-administered questionnaire that has been validated for use in clinical trials or epidemiological studies of sexual function in women.[52] There are sexual function inventories for men that have been published and used within research regarding male sexual function, the International Index of Erectile Function (IIEF)[53] and the Brief Male Sexual Function Inventory (BMSFI).[54] Although these tools have been designed primarily for identifying issues relating to erectile dysfunction more so than psychosocial determinants of sexual changes, they may have relevance in the research setting as part of a variety of assessment tools investigating sexuality changes post-trauma injury.

The Derogatis Sexual Functioning Inventory (DFSI) is another human sexual function inventory for use with both males and females that has been widely used in sexuality research.[55] This is a 254-item questionnaire arranged into 10 subtests: information, experience, drive, attitudes, psychological symptoms, affects, gender role definition, fantasy, body image and sexual satisfaction.[55] The authors state that it can be self-administered by participants regardless of marital status or sexual orientation.[55] It was designed to be utilized as an outcome measure and this combined with the administration of this tool being approximately 60 min or longer limits its use in the clinical setting. However, as a validated multidimensional tool, it does have the potential to be used in individual or multicenter research into sexuality changes for trauma survivors.

Hatzichristou *et al.*,[47] endorse the use of the Brief Sexual Symptom checklist (BSSC) for men and the BSSC for women as well as the Sexual Complaints Screener (SCS) for men and SCS for women as screening tools for the clinical setting developed by the International Consultation in Sexual Medicine.[47] This endorsement is given in the context of sexual medicine; therefore, further research is require to assess if these screening tools are appropriate for the multidisciplinary rehabilitation setting to identify sexuality changes for trauma survivors.

Lastly, the Procurement Management Information System (PROMIS®) Sexual Function and Satisfaction Measures Brief Profile (PROMIS® SexFS) is a customizable research tool that can be self-administered by participants of diverse gender and sexual orientation.[56] While its validity is for use in cancer populations, the measure is intended for use across a broad range of diagnostic groups. The PROMIS® SexFS includes 81 items in 11 domains: interest in sexual activity, lubrication, vaginal discomfort, erectile function, global satisfaction with sex life, orgasm, anal discomfort, therapeutic aids, sexual activities, interfering factors and screener questions.[56] This is a relatively new tool that is currently being tested in other target groups other than cancer, thus may be a tool that has some future validity in the trauma population.

## Limitations

Owing to the lack of research articles for burn and TBI trauma, results must be viewed with caution. For example, there was no evidence from the literature in relation to burns survivors having difficulties with libido/sexual arousal; however, with only a limited number of research articles sourced that focused solely on aspects of sexuality, it is possible that this issue has not been researched adequately within this trauma cohort.

## Conclusion

Trauma injury can have a significant impact on an individual’s sexuality resulting in difficulties in psychosocial adjustment. Although this is an important issue, sexuality has been largely ignored within trauma research and rehabilitation. There is an urgent need for quality empirical research across all trauma groups to develop effective comprehensive therapeutic rehabilitation strategies relating to sexuality in order to facilitate improvements in the QoL of all trauma survivors.

Collaborative research with medical, allied health, and sexuality-based researchers is required to ensure that research includes sexual diversity and broader perspectives of sexuality and acknowledges the differences in sexual response between males and females.

## Footnotes

1 The asterisks signify the truncated search terms entered into the data bases during the literature search.

2 The asterisks signify the truncated search terms entered into the data bases during the literature search.
